# Diagnostic Dilemma in Left Ventricular Noncompaction Cardiomyopathy: Thrombus or Tumor?

**DOI:** 10.7759/cureus.103100

**Published:** 2026-02-06

**Authors:** Mohamed O Elhussain, Mazen Ahmed, Ragda Ali

**Affiliations:** 1 Internal Medicine, Wayne State University Detroit Medical Center, Detroit, USA; 2 Internal Medicine, Corewell Health Dearborn Hospital, Dearborn, USA; 3 Internal Medicine, University of Khartoum, Khartoum, SDN

**Keywords:** contrast echocardiography, intracardiac mass, left ventricular noncompaction, left ventricular thrombus, tumor-thrombus complex

## Abstract

Left ventricular noncompaction cardiomyopathy (LVNC) is associated with thromboembolic complications and can complicate intracardiac mass interpretation. A 58-year-old man with prior left ventricular (LV) apical thrombus presented with abdominal pain and weight loss. Contrast-enhanced transthoracic echocardiogram (TTE) showed severe LV dysfunction (left ventricular ejection fraction (LVEF) 15-20%) and a 1.5 cm mobile apical mass with apparent central contrast uptake, raising concern for a tumor in the setting of suspected prostate malignancy. Heparin was initiated. On day 3, the mass had resolved, and repeat echocardiography demonstrated prominent apical trabeculations and a low-flow apex with findings suggestive of LVNC, indicating that the perceived "vascularity" likely reflected pseudo-enhancement. This case emphasizes serial imaging and multimodality assessment when uncertainty persists.

## Introduction

Left ventricular noncompaction cardiomyopathy (LVNC) is characterized by prominent left ventricular (LV) trabeculations and deep intertrabecular recesses and is associated with heart failure, arrhythmias, and thromboembolic complications [1]. In patients with reduced LV systolic function, blood stasis within the ventricle, particularly near the apex, increases the risk of LV thrombus formation and complicates both diagnosis and management [2].

When an LV apical mass is identified on a transthoracic echocardiogram (TTE), the differential diagnosis includes thrombus, primary cardiac tumor, and metastatic disease. Contrast-enhanced echocardiography (using ultrasound-enhancing agents) is commonly used to improve endocardial border definition and help characterize intracardiac masses because thrombus is typically avascular, whereas tumors may demonstrate enhancement or perfusion [3,4]. However, diagnostic pitfalls can occur in heavily trabeculated ventricles, where low-flow states and contrast pooling within recesses can mimic a discrete mass and suggest a neoplasm.

We report a case in which an LV apical mass with apparent central vascularity raised concern for a primary cardiac tumor or cardiac metastasis in the setting of suspected prostate malignancy, but serial imaging demonstrated rapid resolution after empiric anticoagulation and revealed prominent apical trabeculations consistent with suspected LVNC.

## Case presentation

**Figure 1:**
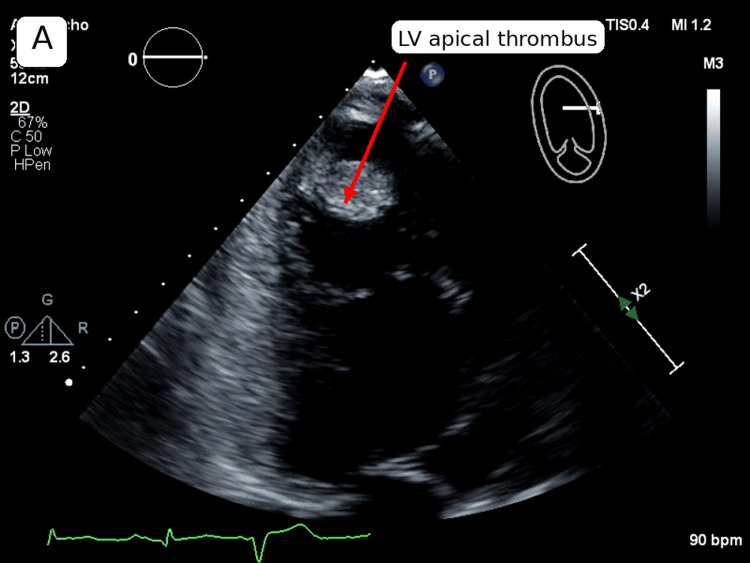
Apical transthoracic echocardiogram demonstrating a mobile left ventricular apical mass (arrow) favored to represent thrombus. In a markedly trabeculated, low-flow apex, adjacent trabeculations and contrast pooling can create a pseudo-mass or pseudo-enhancement appearance that may mimic tumor vascularity on contrast imaging

A 58-year-old man with a history of heart failure with reduced ejection fraction complicated by a remote LV apical thrombus presented on day 1 with diffuse abdominal pain and unintentional weight loss. Initial laboratory testing showed a B-type natriuretic peptide (BNP) of 4,567 pg/mL, consistent with the patient's prior baseline. High-sensitivity troponin was mildly elevated but downtrending (66 ng/L on admission, 51 ng/L on repeat), and the electrocardiogram was unremarkable without acute ischemic changes. Baseline hemoglobin, platelet count, and creatinine were within normal limits. Given his known heart failure and point-of-care ultrasound findings in the emergency department, a formal contrast-enhanced TTE was obtained. The TTE demonstrated recurrent severe LV systolic dysfunction (left ventricular ejection fraction (LVEF) 15-20%) and a 1.5 cm mobile LV apical mass favored to represent thrombus. On contrast imaging, there was an apparent central contrast signal within the lesion (Figure 1), raising concern for a neoplastic process. However, in a highly trabeculated apex with low-flow physiology, contrast pooling within intertrabecular recesses may produce pseudo-enhancement that can mimic internal "vascularity" rather than true tumor perfusion. These findings represented deterioration compared with prior cardiac imaging, as a prior TTE had demonstrated an LVEF of 20-25% with an LV apical thrombus, while subsequent follow-up imaging performed months later showed recovery of LVEF to 50-55% with the interval resolution of the thrombus.

**Figure 2:**
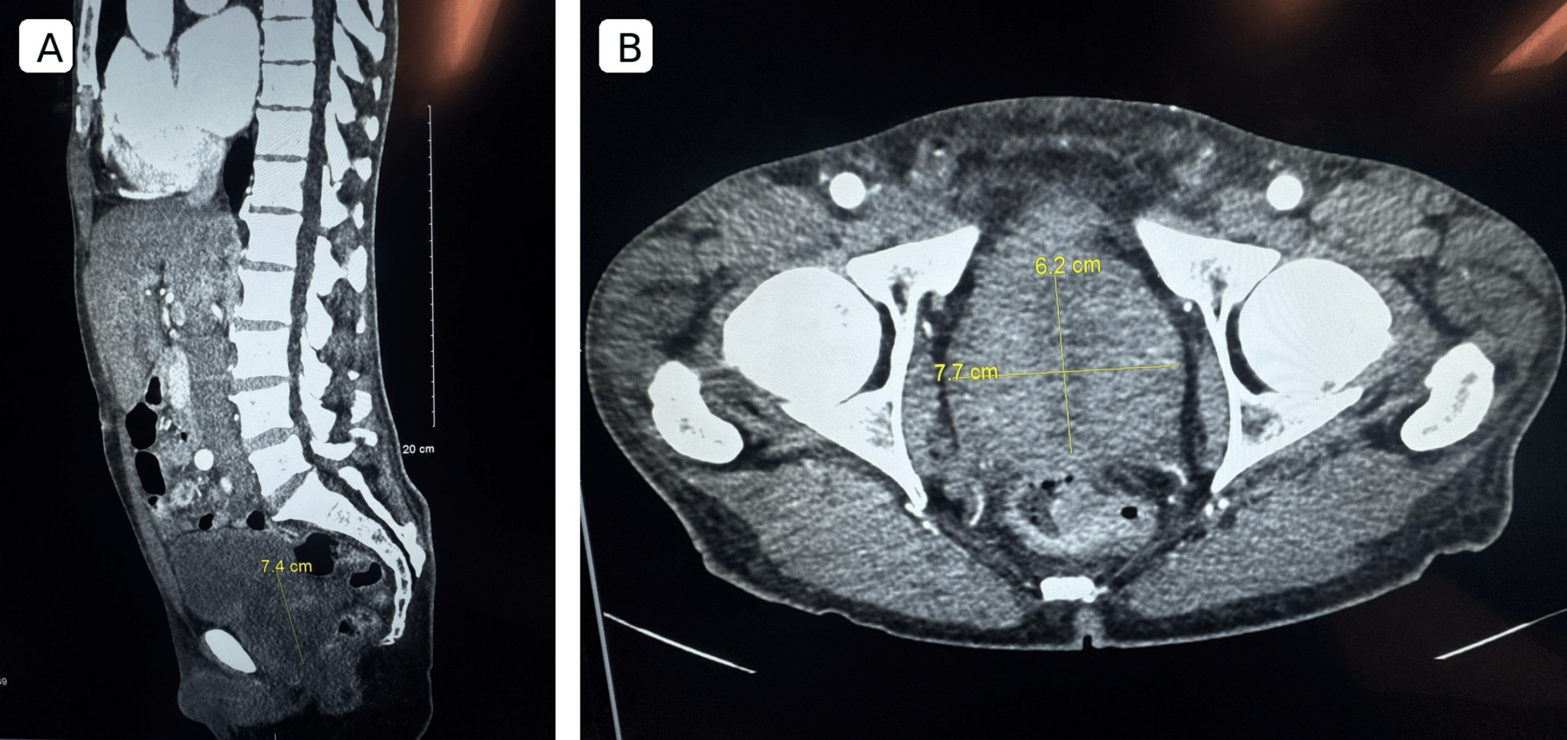
Computed tomography of the abdomen and pelvis obtained during the same admission demonstrating a markedly enlarged, heterogeneous prostate measuring 7.4×6.2×7.7 cm, indenting the bladder base

Given the echocardiographic appearance and the apparent contrast signal, a neoplastic cardiac process was strongly considered. The differential diagnosis included primary cardiac tumor, malignancy-related cardiac involvement or metastasis, and a tumor-thrombus complex, in addition to recurrent LV thrombus. Concern for malignancy was further supported by the patient's history of high-grade prostatic intraepithelial neoplasm and computed tomography (CT) of the abdomen and pelvis during the same admission demonstrating a markedly enlarged heterogeneous prostate (7.4×6.2×7.7 cm) indenting the bladder floor (Figure 2). The patient was started on an intravenous heparin infusion due to thromboembolic risk, while an oncology evaluation was initiated.

**Figure 3:**
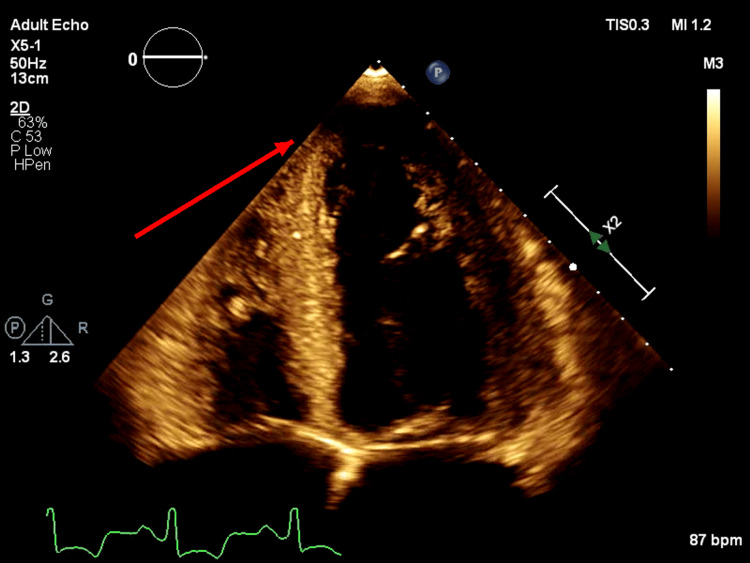
Repeat transthoracic echocardiography demonstrating the interval resolution of the left ventricular apical thrombus with prominent apical trabeculations

On day 3, repeat cardiac imaging demonstrated interval non-visualization of the previously seen LV apical mass (Figure 3). Bedside echocardiographic reassessment did not identify a discrete apical thrombus and instead demonstrated prominent apical trabeculations with apical stasis, raising suspicion for findings suggestive of LVNC with a low-flow apex. A comprehensive TTE performed later the same day similarly showed that the LV apical mass seen on day 1 was no longer present. While this interval change was compatible with thrombus response to anticoagulation, embolization is an alternative explanation for the disappearance, although the patient did not develop new focal neurologic deficits or other clinical evidence of systemic embolization during hospitalization. LVNC was not formally confirmed during this admission with dedicated echocardiographic criteria or cardiac magnetic resonance imaging, and the diagnosis remained suggestive based on the echocardiographic appearance. Given his prior LV thrombus, recurrent severe LV systolic dysfunction, and evidence of apical stasis, lifelong anticoagulation was recommended at discharge, and the patient was discharged on rivaroxaban 20 mg daily.

## Discussion

Differential diagnosis of an LV apical mass

A new LV apical mass on TTE in a patient with severe systolic dysfunction most commonly represents thrombus due to blood stasis, particularly when there is apical akinesis or a low-flow apex [2]. However, the differential diagnosis is broad and includes primary cardiac tumors and metastatic disease; importantly, cardiac metastases are more common than primary cardiac tumors [5,6]. In our case, the initial TTE appearance of a discrete apical mass raised immediate concern for a true intracardiac lesion, and the concurrent systemic symptoms increased the need to consider neoplasm alongside thrombus.

Contrast echocardiography: strength and key pitfall

Contrast-enhanced echocardiography (using ultrasound-enhancing agents) improves endocardial border definition and is commonly used to help characterize intracardiac masses [3]. Perfusion patterns can aid differentiation because thrombi are typically avascular, whereas tumors may demonstrate enhancement or perfusion [4]. In this patient, apparent central vascularity increased suspicion for neoplasm and prompted oncologic evaluation. Nevertheless, contrast interpretation can be confounded by anatomic and flow-related factors, particularly in heavily trabeculated ventricles and low-flow apical states, where contrast pooling within recesses can create pseudo-mass effects and mimic enhancement. The rapid interval non-visualization of the mass on repeat imaging after the initiation of heparin, together with bedside identification of prominent apical trabeculations and reduced apical velocities, was most consistent with the interval resolution of thrombus in response to anticoagulation and/or a trabeculation-related mimic rather than a fixed neoplasm. Embolization is an alternative explanation for the interval disappearance and was assessed clinically and or radiologically as described above.

LVNC as an underlying substrate

LVNC is characterized by prominent trabeculations and deep intertrabecular recesses, most commonly involving the LV apex, and is associated with heart failure, arrhythmias, and thromboembolic events [1]. In this case, bedside findings of marked apical trabeculations and apical low-flow physiology suggested an LVNC phenotype and provided a unifying explanation when the echocardiographic impression was discordant. Cardiac magnetic resonance imaging was not performed during this admission, which limits definitive confirmation; further outpatient evaluation was planned.

Anticoagulation considerations in suspected LVNC with low-flow apex

Anticoagulation decisions in LVNC are individualized, but higher-risk features such as prior thromboembolism, documented LV thrombus, atrial fibrillation, and significant LV systolic dysfunction generally support long-term anticoagulation [1,2]. In our patient, the prior LV thrombus, recurrent severe LV systolic dysfunction, and evidence of apical stasis supported the decision for lifelong anticoagulation. The comprehensive TTE on 07/10/2025 demonstrating interval non-visualization of the previously seen LV apical mass was compatible with interval resolution consistent with anticoagulation response, supporting thrombosis as the most likely mechanism despite the initial concern for malignancy.

Clinical lessons

An LV apical "mass" with apparent vascularity on contrast echocardiography does not exclude thrombus or trabeculation-related pseudo-mass, especially in suspected LVNC where recesses and low-flow physiology can mimic solid lesions. Serial imaging can be decisive, and when diagnostic uncertainty persists, multimodality assessment should be considered to clarify the etiology of an apparent intracardiac mass.

## Conclusions

An LV apical mass with apparent vascularity on contrast-enhanced echocardiography can raise concern for a primary cardiac tumor or metastatic disease, particularly when systemic symptoms and a concurrent malignancy workup are present. In patients with severe LV dysfunction and prominent apical trabeculations, however, low-flow physiology and contrast pooling within trabecular recesses may mimic a neoplastic mass. Rapid interval non-visualization on serial echocardiography after anticoagulation was compatible with interval resolution consistent with anticoagulation response and shifted suspicion toward an LVNC phenotype with a trabeculated, low-flow apex as the underlying substrate. Serial imaging should be considered when echocardiographic findings are discordant, and long-term anticoagulation may be warranted in high-risk patients with recurrent thrombus and persistent apical stasis.
